# Acute Appendicitis: Is Removal of a Normal Appendix Still Existing and Can We Reduce Its Rate?

**DOI:** 10.4103/1319-3767.51367

**Published:** 2009-07

**Authors:** Gamal Khairy

**Affiliations:** Division of General Surgery, Department of Surgery, PO Box 7805, Riyadh - 11472, Saudi Arabia

**Keywords:** Acute appendicitis, laparoscopy, computed tomography

## Abstract

**Background/Aim:**

To determine the incidence of negative appendectomies and to identify factors that may reduce the risk of having the normal appendices removed surgically.

**Design:**

Cross-sectional study.

**Setting:**

College of Medicine and King Khalid University Hospital, Riyadh, Saudi Arabia.

**Materials and Methods:**

The surgical and histological data of 852 patients who underwent appendicectomy were reviewed. All incidental or interval appendicectomies were excluded. Only patients who were admitted and whose appendices were removed and subjected to histology were included (585 patients). The data on patients who had a normal appendix on histology further analyzed to include demographics, specific investigations, operative findings of the appendix and additional operative findings that need other surgical procedures.

**Results:**

A normal appendix was removed in 54 (9.2%) of the patients. Only 5.5% of those patients had a computed tomography (CT) scan preoperatively and 3.7% had diagnostic laparoscopy. In 21 patients, additional operative and histological findings were obtained that might have caused the right lower abdominal pain.

**Conclusion:**

In spite of the advances in the diagnostic and imaging techniques, the rates of negative findings on appendicectomy have not decreased much. Clinical judgment is still the most important factor in the management of patients with suspected acute appendicitis. The routine use of CT scan or diagnostic laparoscopy for all patients who are suspected to have appendicitis is neither cost-effective nor safe.

Appendectomy remains the most frequently performed emergency abdominal surgical procedure.[1] The lifetime risk of acute appendicitis for men and women is 8.6% and 6.7%, respectively. However, the lifetime risk of having an appendectomy is 12% for men and 25% for women.[2–4]

Appendicitis remains a difficult diagnosis,[5] and the most accurate means of its diagnosis remains a source of debate. Several diagnostic tools and scoring systems to diagnose early appendicitis have been developed, characterized as noninvasive, understandable and cost effective.[6,7] It is imperative that patients with appendicitis go to the operating room early as there is a significant increase in the morbidity and mortality in those experiencing appendiceal rupture.[8–12] This has led to 10–30% of the normal appendices being removed at open operation.[2,13–15] The cost to both the patient and the health care system of those so-called “negative appendicectomies” (NAs) is considerable[2,16,17] and a complication rate of up to 6.1% following removal of normal appendices was reported.[18] The use of laparoscopy did not reduce the rate of NA.[19] The aim of this study is to determine the incidence of negative appendectomies in our practice and to identify factors that may reduce the risk of having the normal appendices removed surgically.

## MATERIALS AND METHODS

A retrospective chart analysis was performed for all the patients who underwent appendectomy at the King Khalid University Hospital, Riyadh, in the period 1998-2003. All incidental and interval appendectomies were excluded. Only patients who were admitted for suspected acute appendicitis and whose appendices were physically removed and subjected to histology were included. The appendicectomy was carried out using either the standard or the modified gridiron incision. When there was a discrepancy between the surgeon's operative diagnosis and the pathologist's diagnosis, based on gross and histological examination of the appendix, the pathologist's diagnosis was assumed to be correct. Acute appendicitis was diagnosed on histological grounds according to the following criteria: Macroscopic signs include intravascular injection of serosa, fibrinous and purulent film, edematous, necrotic changes of the wall and blood or pus on opening the appendix. Microscopic signs include focal or expanded erosion, ulceration, abscess, fistula and necrosis or perforation. The data of patients who had normal appendix on histology were analyzed with regard to demographics (e.g., age, sex), specific investigation (preop computed tomography [CT], diagnostic laparoscopy), operative finding (of the appendix), additional operative and histological pathology and other surgical procedures needed to be performed.

## RESULTS

Out of the 852 patients who were reviewed, 585 patients were found to be eligible for entry in the study. Table 1 shows the histopathological results of patients who underwent appendicectomy. A normal appendix was removed in 54 (9.2%) patients, 39 women (72%) and 15 men (27.2%). The mean age of those who had normal appendices was 23 + 8.67 years (range 12-60 years). Only three (5.5%) of those patients had a CT scan preoperatively and two (3.7%) had diagnostic laparoscopy. At operation, the surgeons considered 11 of the 54 normal appendices to be acutely inflamed. In 21 patients, additional operative and histological findings were obtained that might have caused the right lower abdominal pain and treated if necessary [Table 2]. In six patients (11%), the underlying cause needed operative intervention [Table 2].

**Table 1:** The histopathology results of patients who underwent appendectomy

Histopathology	Patients	
	
	*n*	%
Normal (a)	54	9.2
Acutely inflamed (b)	69	11.8
Suppurative (c)	370	63.2
Perforated (d)	52	8.9
Others (e)	40	6.9
Total	585	100

(a) Normal: No evidence of inflammation

(b) Acutely inflamed: Microscopic evidence of inflammation

(c) Suppurative/gangrenous: Macroscopically inflamed with periappendiceal pus or gangrene

(d) Perforated: Perforation of the appendix with generalized or localized peritonitis

(e) Others: Included carcinoid tumor of the appendix, adenocarcinoma, endometriosis,… etc

**Table 2:** Other operative diagnoses obtained in patients with normal appendix

Diagnosis	*n*	Treatment (*n*)
Ovulation bleeding	6	
Ovarian cyst	5	Ovariectomy (2)
Fecolith	2	
Torsion of appendices epiploicae	2	Excision (2)
Mesenteric adenitis	2	
Adhesions	2	Adhesolysis (1)
Uterine fibroid	1	
Caecal nodule	1	Excision + oversewing (1)

## DISCUSSION

The diagnosis of appendicitis is not always straight forward. Approximately 20-33% of the patients suspected of having acute appendicitis present with atypical findings.[20,21] The indication for operation is usually based on a combination of clinical and laboratory findings.[22–24] The important aspect of this diagnostic dilemma is the fear of perforated appendicitis, which can lead to increased morbidity and prolonged hospital stay. Traditionally, the most effective way to decrease the rate of perforation is to have a lower threshold for taking the patient to the operating room at the expense of increasing the negative appendectomy rate.[25]

The overall NA rate in the present series is 9.2%, which is comparable with previously reported rates elsewhere.[26–28] However, some recent studies reported rates between 15% and 35%.[29–32] More than 70% of our patients who had NA were females and their mean age was 23 years + 8.67. The findings are in line with the reported difficulties involved in making the correct diagnosis in females.[33] Similarly, others confirmed that the incidence of misdiagnosis increased for women of reproductive age.[34] Accordingly, some investigators advised routine diagnostic laparoscopy in women of child-bearing age with suspected appendicitis, but in men its use is not recommended.[35,36] However, in a recent publication, Ekeh *et al*.[19] concluded that laparoscopic appendicectomy was associated with an increased rate of NA.

In the present series, the surgeon considered 11 of the 43 patients with NA to have acute appendicitis. Such disagreement between the surgeon and the pathologist was reported before.[37] Also, 5.5% of our patients had NA in spite of having a preoperative CT scan. This diagnostic tool has not been shown conclusively to improve the outcome in terms of negative findings on appendicectomy and complicated appendicitis.[38–39] One of the earliest studies supporting the use of routine appendiceal CT was published by Rao *et al.* in 1998,[40] who concluded that routine appendiceal CT should be performed to reduce the use of hospital resources. A follow-up study by the same research group demonstrated a decrease in the NA rate from 20% to 7%.[41] Many studies that have been published since then do not support the liberal use of CT scan in the diagnosis of appendicitis. Perez *et al.* showed no improvement in the NA with the increased use of CT.[42] Clinical assessment without radiological imaging was shown to be superior and patients went to the operative room in a shorter time than those having preoperative CT.[43] However, some recent publications[44–45] show the significant benefit of using a preoperative CT scan in reducing the rate of NA.

In the current series, 3.7% of those who had NA underwent diagnostic laparoscopy. Some previous reports showed that the use of laparoscopy improved the accuracy of diagnosis in acute appendicitis. The incidence rate of removing a normal appendix has been reduced to 8-20% in those patients undergoing the laparoscopic procedure[46–47] compared with 10-33% in patients undergoing an open procedure.[48–49] Others reported a further lower NA rate for laparoscopic appendicectomy (4-13%), claiming that a normal appendix can be safely left in place.[50–52] However, such a policy may expose the patient to potentially harmful investigation and risks missing the diagnosis of an early appendicitis.[53] Others advocated the removal of the normal-appearing appendix because at histopathology examination the normal-appearing appendix might show increased cytokines, indicating an inflammatory response.[54]

In conclusion, in spite of the advances in the diagnostic and imaging techniques, the rates of the negative findings on appendicectomy have not decreased much. Clinical judgment is still the most important factor in the management of patients with suspected acute appendicitis. The routine use of CT scan or diagnostic laparoscopy for all patients who are suspected to have acute appendicitis is neither cost-effective nor safe. However, the use of these two diagnostic procedures in selected controversial cases can enhance the accuracy of diagnosis, reduce the cost and reduce the rate of NA.
